# Genetic tool for fate mapping of Oct4 (Pou5f1)-expressing cells and their progeny past the pluripotency stage

**DOI:** 10.1186/s13287-019-1520-6

**Published:** 2019-12-16

**Authors:** Andrey A. Kuzmin, Veronika V. Ermakova, Sergey A. Sinenko, Sergey V. Ponomartsev, Tatiana Y. Starkova, Elena V. Skvortsova, Olga Cherepanova, Alexey N. Tomilin

**Affiliations:** 1 Institute of Cytology, Russian Academy of Sciences, St. Petersburg, Russian Federation; 2 Cleveland Clinic, Lerner Research Institute, Cleveland, OH USA; 3 St. Petersburg State University, St. Petersburg, Russian Federation

**Keywords:** Molecular sensor, *Oct4*, *Pou5f1*, Cre-loxP, FlpO-FRT, *Rosa26*, Genome editing

## Abstract

**Background:**

Methods based on site-specific recombinases are widely used in studying gene activities in vivo and in vitro. In these studies, constitutively active or inducible variants of these recombinases are expressed under the control of either lineage-specific or ubiquitous promoters. However, there is a need for more advanced schemes that combine these features with possibilities to choose a time point from which lineage tracing starts in an autonomous fashion. For example, the key mammalian germline gatekeeper gene *Oct4 (Pou5f1)* is expressed in the peri-implantation epiblast which gives rise to all cells within embryos. Thus the above techniques are hardly applicable to *Oct4* tracing past the epiblast stage, and the establishment of genetic tools addressing such a limitation is a highly relevant pursuit.

**Methods:**

The CRISPR/Cas9 tool was used to manipulate the genome of mouse embryonic stem cells (ESCs), and various cell culture technics—to maintain and differentiate ESCs to neural cell, lentivirus-based reprogramming technique—to generate induced pluripotent stem cells (iPSCs).

**Results:**

In this paper, we have developed a two-component genetic system (referred to as O4S) that allows tracing Oct4 gene activity past the epiblast stage of development. The first component represents a knock-in of an ubiquitous promoter-driven inducible Cre, serving as a stop signal for downstream tdTomato. Upon activation of Cre activity with 4-hydroxytamoxifen (4-OHT) at any given time point, the recombinase excises a stop signal and poses the second component of the system—the FlpO recombinase, knocked into 3’UTR of *Oct4,* to be expressed upon activation of the latter gene. *Oct4*-driven expression of FlpO, in turn, triggers the tdTomato expression and thus, permanently marks Oct4^+^ cells and their progeny. We have validated the O4S system in cultured ESCs and shown that it is capable, for example, to timely capture an activation of *Oct4* gene during the reprogramming of somatic cells into iPSCs.

**Conclusions:**

The developed O4S system can be used to detect *Oct4* activation event, both permanent and transient, in somatic cell types outside the germline. The approach can be equally adjusted to other genes, provided the first component of the system is placed under transcriptional control of these genes, thus, making it a valuable tool for cell fate mapping in mice.

## Background

Lineage tracing is one of the most comprehensive methods of developmental biology. The method often takes advantage of site-specific recombinases, most commonly Cre, FlpO, and Dre [1–3]. These recombinases, placed in the context of the regulatory elements of a chosen gene, work in tandem with ubiquitous promoter-driven reporter genes, whose expression is blocked by an upstream loxP/FRT/Rox-flanked STOP-signal (i.e., multiplied polyadenylation sites). When gene of interest is turned on, de novo expressed recombinases excise the STOP-signal, triggering constitutive expression of the reporter gene, thereby, permanently marking the cells and their progeny. However, the described approach is not suitable for genes that have complex expression patterns [4]. To address this issue, inducible Cre systems such as one fused to the estrogen receptor ligand-binding domain (ERT) have been developed, providing an opportunity to set up reporter gene expression in a combined cell type- and hydroxytamoxifen (4-OHT, a ligand for the ERT)-specific fashion [5]. This approach, however, has its limitation and is hardly applicable, for example, for discovering novel spatiotemporal expression patterns of genes.

The transcription factor Oct4 was isolated and characterized in the late 1980s–early 1990s [6, 7]. This octamer-binding protein containing the DNA-binding POU-domain is necessary for the maintenance of pluripotency of the epiblast and its cultured counterpart, embryonic stem cells (ESCs) [8–10]. Besides being expressed in the epiblast, Oct4 expression has been shown to be associated with and required for primordial germ cells, or PGCs [11, 12]. The feature underlies Oct4 capability to serve as one of the factors to reprogram somatic cells into induced pluripotent stem cells (iPSCs) that Oct4 belongs to the so-called pioneer transcription factors [13, 14]. Within the concept of pioneer factor activity, Oct4 can be viewed as a factor capable to promote cellular plasticity in general, not necessarily leading to the establishment of pluripotent cell state [15–17]. Indeed, our recent genetic studies have provided compelling genetic evidences that Oct4 is required for switching of the smooth muscle cells (SMCs) phenotype within atherosclerotic plaques—a process considered to be associated with some de-differentiation, i.e., with the acquisition of cellular plasticity [18]. This is perhaps the first example of natural cell plasticity dependent upon Oct4 function.

Albeit Oct4 is postulated to have no role in adult mice [19], in the context of certain pathologies it might have an important function, and the one recently described in SMCs of atherosclerotic plaques [18] is possibly not unique in the adult organism. To identify other cell types, tissues, and organs, which could possibly engage Oct4 function, both in the context of homeostasis and pathologies, we have developed a novel reporter system. This two-component system allows detection of de novo *Oct4* (*Pou5f1*) expression in cells that have passed the pluripotent stage. The generated and tested in embryonic stem cells (ESCs) genetic tool might be used to discover novel functions of *Oct4* and, after some modifications, of other genes in both cultured cells and mice.

## Methods

### Plasmids

For CRISPR/Cas9-mediated knock-in, the pX330-U6-Chimeric_BB-CBh-hSpCas9 plasmid with additional fluorescent markers (mCherry or EGFP) was used. gRNAs were designed using the Benchling online platform and cloned into BbsI sites of the aforementioned plasmid. As a backbone for the creation of the plasmid Rosa26-TRE-CAG-Frt(Ert2CreErt2-STOP)Frt-tdTomato-PGKneo was used Ai65(RCFL-tdT) targeting vector (Addgene plasmid #61577). As a source of Ert2CreErt2 and FlpO recombinases, the Addgene plasmids #13777 and #13793 respectively were used. For reprogramming experiments, lentiviral plasmids M2rtTA and pHAGE2-OKSM were used [20].

### Cell culture

Unless specified, all cell culture products were from ThermoFisher Scientific (Gibco). Murine E14 Tg2a ESCs (BayGenomics) cells were passaged using 0.05% Trypsin-0.01% EDTA solution under standard feeder-free conditions on gelatinized tissue culture dishes in mouse ESC media: knockout DMEM supplemented with 15% ES cell-qualified fetal bovine serum, 100 U/ml penicillin, 100 μg/ml streptomycin, 2 mM L-glutamine, 1 × non-essential amino acids, 50 μM β-mercaptoethanol, and 500 U/ml of bacterially expressed in-house hLIF. For reprogramming experiments standard N2B27 medium (Gibco) was used, supplemented with 500 U/ml hLIF, 3 μM GSK3 inhibitor CHIR99021 (Axon), and 1 μM MEK inhibitor PD0325901 (Axon), and 3 μg/ml doxycycline. Neural differentiation was performed as described previously with minor changes [21, 22].

### Teratoma formation assay

Mouse O4S ESC growing on gelatin-coated dishes in ESC medium, were harvested with 0.05% Trypsin-0.01% EDTA (Gibco), resuspended in PBS and injected subcutaneously (1 × 10^6^ cells) into athymic CD-1 NUDE mice. Four animals with two injections per mouse were used for experiments. After 4–6 weeks teratomas were removed from euthanized animals and processed for histological analysis.

### Preparation of sections for histological analysis

Teratomas were excised, washed in PBS, and fixed in 4% PFA at 4 °C overnight. Specimens were dehydrated in an ethanol series (70–80–96%) and isobutanol:paraffin series (2:1–1:1–1:2) then embedded in paraffin (Sigma). For each teratoma, blocks 5 × 5 mm were used for analysis until all embryonic lineages were identified. Ten 7-μm sections with 30-μm steps were prepared for each block using Leitz 1208 microtome (Germany). Paraffin sections were washed in xylene, rehydrated through an ethanol series (100–70%), and washed with water. Next, sections were incubated in hematoxylin for 5 min, washed with water and incubated with eosin for 1 min. After washing and dehydration, sections were mounted in Canada balsam for further analysis.

### Transient transfection

Transient transfection experiments were performed in tissue culture plates using the FuGene HD transfection reagent (Promega) in the mouse ESC cell media (see above) supplemented with 100 U/ml penicillin, 100 μg/ml streptomycin, and 500 U/ml hLIF. Cells were seeded at a density of 5 × × 10^3^/cm^2^ per well of 24-well plates. The next day, the media was changed to OptiMEM, and 2 h later, the transfection mix (1 μg plasmid, 2 μl FuGene HD, and 250 μl OptiMEM) was added into the wells. After 12 more hours, the media was changed to standard mouse ESC medium, and after an additional 24 h, replaced with the same medium containing antibiotics.

### Genome editing

For performing a knock-in, the following sgRNA sequences were picked: 5′-GTCTCCCATGCATTCAAACTG-3′ for *Pou5f1* gene and 5′- ACTCCAGTCTTTCTAGAAGA-3′ for *Rosa26* gene. Firstly, *Pou5f1* was edited by transfection of the cells with pX330-U6-Chimeric_BB-CBh-hSpCas9 bearing *Pou5f1* sgRNA sequence and Oct4-FlpO plasmids using FuGene HD transfection reagent. After selection on 1 μg/ml puromycin (Sigma) for 4 days, obtained clones were genotyped and proceeded to editing in *Rosa26* locus by transfection of the cells with pX330-U6-Chimeric_BB-CBh-hSpCas9 bearing *Rosa26* sgRNA sequence and Rosa26-Cre plasmids. Following 1 week of selection at 500 μg/ml G418, clones were picked, genotyped, and sequenced to confirm a correct genome editing in both alleles. Off-target analysis was performed via sequencing of amplicons flanked potential off-target sites, predicted by Benchling online-software.

### RNA isolation and RT-PCR

Total RNA from cells was isolated from cells by TRIzol Reagent (Invitrogen). For cDNA synthesis, 2–5 μg of total RNA was used. cDNA was synthesized using M-MuLV Reverse Transcriptase (Thermo Scientific) and oligo(dT) primer (Thermo Scientific). Quantitative RT-PCR was performed using 5× qPCRmix-HS SYBR buffer (Evrogen), using the Bio-Rad CFX-96. Expression levels were normalized to endogenous GAPDH RNA using Bio-Rad CSX manager software.

### Lentivirus packaging and iPSC generation

For lentivirus packaging, HEK293T cells were transiently transfected using polyethyleneimine with envelope-encoding pMD2G, packaging psPAX2, activator plasmid M2rtTA, or pHAGE2-OKSM plasmid, carrying reprogramming factors (Oct4, Klf4, Sox2, cMyc) [20]. Lentiviruses in cell culture supernatant were collected and concentrated as described elsewhere [23–27]. Differentiated mouse NSCs were seeded (20 × 10^3^ cells per well) on poly-L-ornithine (15 μg/ml) and fibronectin (10 μg/ml) coated 24-well plate in NSC medium (see above). The next day, the medium was replaced to fresh NSC and cells were infected overnight with the lentiviruses M2rtTA and pHAGE2-OKSM. The next day, the medium was changed to NSC medium containing 3 μg/ml Dox. Media was changed every day and on the day 3 following Dox addition cells were replated onto wells of 6- well plates pre-coated with gelatin and feeder cells (mitomycin C-treated mouse embryonic fibroblasts, MEFs) and cultured in N2B27 media described above. The medium was changed every next or second day and cells were visualized using the EVOS™ FL Auto Imaging System until day 12.

## Results

### Design of the system

**Fig. 1:**
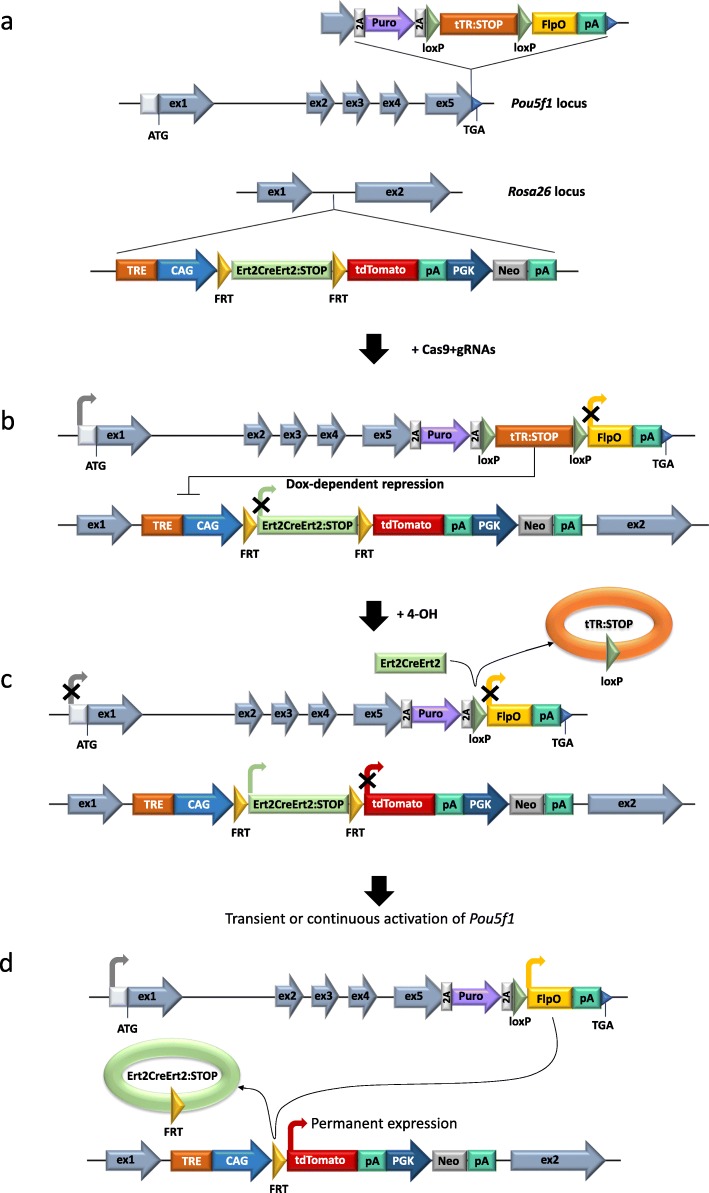
Design of the developed Oct4 tracing system. **a** Indicated cassettes were knocked into *Oct4* (*Pou5f1*) and *Rosa26* loci, using the CRISPR/Cas9 system. **b** *Pou5f1* and *Rosa26* loci after targeting with the above constructs. While *Pou5f1* is constitutively active, tTR blocks expression from CAG-promoter in *Rosa26* locus. This repression can be reversed by doxycycline (Dox) addition. **c** After the addition of 4-hydroxytamoxifen (4-OHT) to the cells of interest, derived from O4S ESCs, tTR:STOP cassette is excised by the Ert2CreErt2 recombinase. Expression of the FlpO recombinase is allowed from this time point. **d** When *Oct4* gene becomes active, FlpO-mediated deletion of the Ert2CreErt2:STOP sequence occurs, resulting in cells permanently labeled with tdTomato regardless of subsequent *Oct4* activity

We set to generate a reporter system that would allow to permanently label non-epiblast cells which have transiently expressed *Oct4* (*Pou5f1*) gene, as well as cells derived thereof. However, because all cells of the adult mouse are derived from Oct4-expressing epiblast, we have developed a conditional reporter that would be converted to Oct4 tracer past the epiblast stage in an inducible manner. To this end, we have designed two genetic cassettes, the Oct4-Puro-2a-Floxed(tTR-STOP)-FlpO and Rosa26-TRE-CAG-Frt(Ert2CreErt2-STOP)Frt-tdTomato-PGKneo (Fig. 1a, b). In the latter cassette, tdTomato expression was set to be triggered by the removal of the upstream STOP sequence with the FlpO recombinase [28]. This recombinase, in turn, is placed under transcriptional control of *Oct4* gene, but remains inactive until triggered in an inducible fashion (Fig. 1c). The second cassette is knocked into the *Rosa26* locus, which is constitutively and ubiquitously active, ensuring that it will reliably capture an onset of *Oct4* expression [29, 30]. To be able to set the above reporter system in a steady position after cells proceed through the pluripotent state in culture or through the epiblast stage during the development (3.5–6.5 dpc), we have additionally introduced an inducible Ert2CreErt2 recombinase, a “leak-proof” version of CreERT [31]. Administration of 4-OHT would then activate Ert2CreErt2 and delete the STOP cassette before FlpO, sensitizing cells to detection of Oct4 expression from the time point on (Fig. 1c). Additionally, tTR repressor-encoding sequence and Tet-response element (TRE) [32] were introduced in the context of targeted *Oct4* and *Rosa26* alleles, respectively, to exclude the possibility of Ert2CreErt2 leakage.

The action of the system can be divided into three stages. During the first stage, the system is supposed to bypass pluripotency stage, being unresponsive to *Oct4* gene activation (Fig. 1b). In the second stage, the system is to put into position ready to record the onset of *Oct4* expression (Fig. 1c). Finally, in the third stage, cells showing the onset permanent or transient *Oct4* expression, as well as the progeny of these cells, are permanently labeled for subsequent detection in vitro or in vivo (Fig. 1d).

### Gene targeting in ESCs

**Fig. 2:**
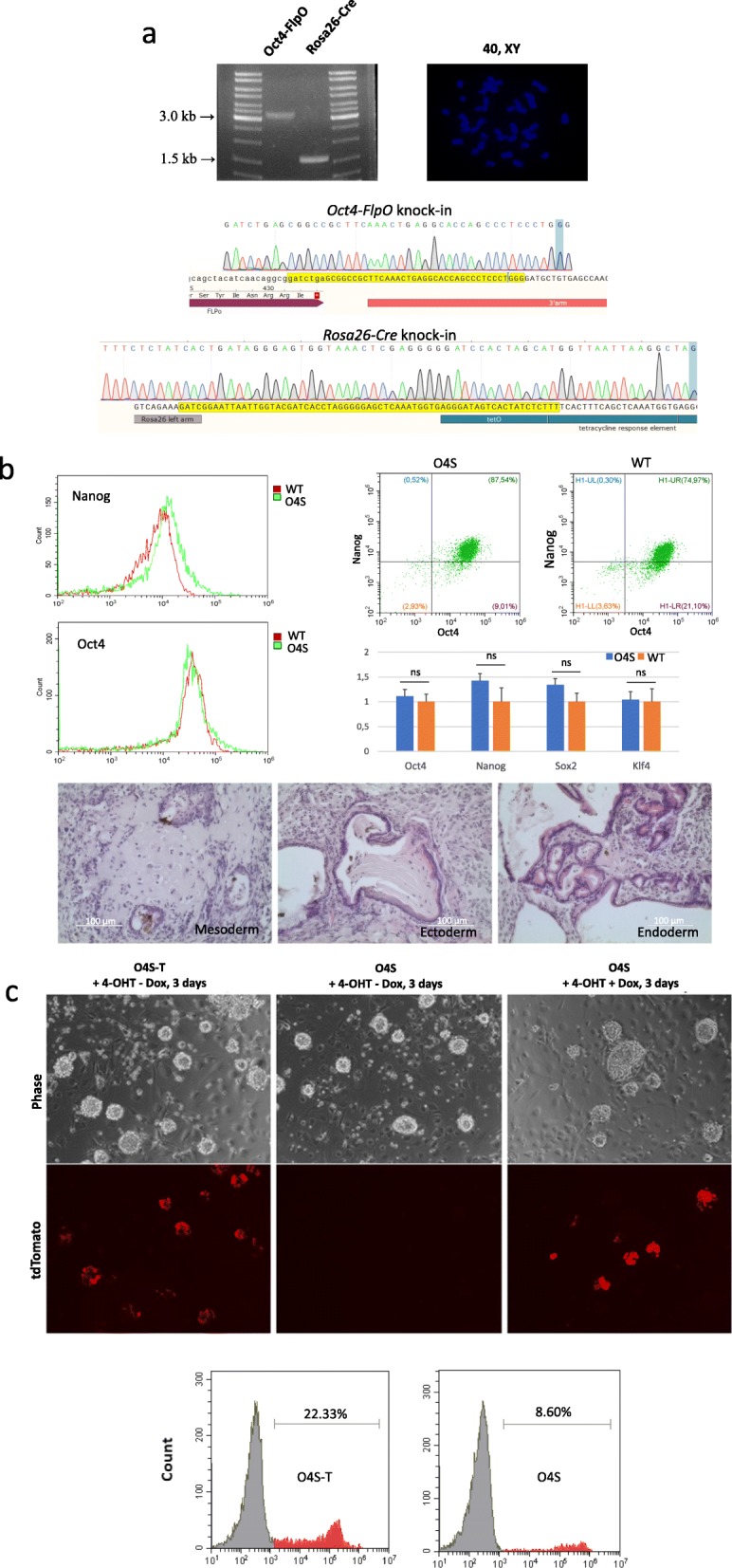
Selection and primary tests of mouse ESCs bearing the developed Oct4 tracing system (O4S ESCs). **a** PCR-genotyping of O4S ESC line showing that constructs were inserted in the correct genome locations (left panel); normal chromosome counts of O4S ESCs (right panel); results of the sequencing of targeted loci, showing the correct position of the inserted cassette (bottom panels). **b** Verification of pluripotency status of the generated O4S ESCs. Comparison of the pluripotency marker expression in O4S vs. wild-type (WT) ESCs by flow cytometry (left and top panels) and qRT-PCR (chart); results are expressed as mean ± SD. Teratoma assay showing the ability of O4S ESCs to differentiate into three germ layers (bottom panels). **c** The ability O4S ESCs to activate the tdTomato expression upon the administration of both 4-OHT and Dox. Representative flow cytometry showing distinct ability to activate tdTomato expression in O4S vs. O4S-tTR ESCs which are different only the tTR-encoding sequence in the targeted Oct4 locus

The assembled cassettes surrounded by respective arms of homology were knocked into the *Oct4* and *Rosa26* loci, using the CRISPR/Cas9 system (Fig. 1a) [33]. The *Oct4* locus was targeted just upstream of the stop codon of the open reading frame. Simultaneous expression was achieved by adding the “self-cleavage” 2A sites [34] in between sequences encoding Oct4, Puromycin N-acetyltransferase (Puro), and tTR proteins (Fig. 1a). For knock-in into the *Rosa26* locus, we used conventional homologous arms, spanning the sequence between the first and second introns. The synthetic CAG promoter was chosen to drive the expression of the cassette as the strongest and ubiquitous in both in vitro and in vivo studies [29, 30]. To reduce the possibility of spontaneous recombination during the transfection process, the constructs were knocked into *Oct4* and *Rosa26 loci* sequentially. Following selection with antibiotics, the obtained clones were genotyped and sequenced to validate the presence of the insert in the desired loci (Fig. 2a). Also, chromosomes were counted in the metaphase spreads of the resulting clones to confirm overall karyotype integrity of the doubly targeted ESC clones, referred hereafter to as O4S, after Oct4 Sensor (Fig. 2a).

Quantitative RT-PCR analysis, flow cytometry, and teratoma assay—all affirmed the retention of the pluripotent state of the O4S ESCs (Fig. 2b). Also, due to strong sequence homologies of the used *Oct4* gRNA within mouse genome, we performed an off-target analysis. Only with the usage of Cas9 nickase it was possible to produce ESC clones with no off-target effects (Additional file 1a). The same analysis of *Rosa26* gRNA off-targets showed no off-target effects even when fully active Cas9 was used (Additional file 1b, Additional file 2).

Primary tests showed that in the absence of Dox and 4-OHT, O4S ESCs remained tdTomato-negative despite very high activity of *Oct4* gene in ESCs. The tTR repressor was introduced into the system to suppress the CAG promoter and to prevent thereby a possible “leakage” of the Ert2CreErt2. To assess whether this was the case, O4S ESCs were compared to those that were identical to the O4S except that they had no tTR sequence downstream of *Oct4* (O4S-T ESCs). Indeed, the absence of Dox (a stabilized Tet analog) along with the presence of 4-OHT completely prevented CAG-driven expression from *Rosa26* locus in the O4S ESCs (compare left and middle panels in Fig. 2c). However, the additional feature also reduced the ratio of tdTomato-positive O4S ESCs after the exposure to Dox (compare left and right panels in Fig. 2c). This might be due to high expression levels of Oct4 (and tTR) in O4S ESCs, causing over-repression of CAG promoter. However, we anticipate that this side effect will not persist in cells with low levels of Oct4 expression, such as SMCs within atherosclerotic lesions [18] and, presumably, other somatic cell types to be discovered.

### System validation

We next set to validate the developed system in vitro by modeling the in vivo situation when the cells were to proceed through the epiblast stage unresponsive to *Oct4* gene status and, after sensitization, becoming responsive to a re-activation of this gene. To this end, the doubly targeted O4S ESCs, considered to be cultured counterparts of the pre-implantation epiblast, were first differentiated, then sensitized and subsequently reprogrammed into the iPSCs. We have chosen differentiation into neural stem-like cells because it is a rather uniform and robust type of differentiation. The efficiency of the differentiation was confirmed by qPCR and immunocytochemistry, which showed a complete downregulation of Oct4 and the presence of the neural stem cell markers Sox2 and Nestin (Additional file 3a) [35].

**Fig. 3:**
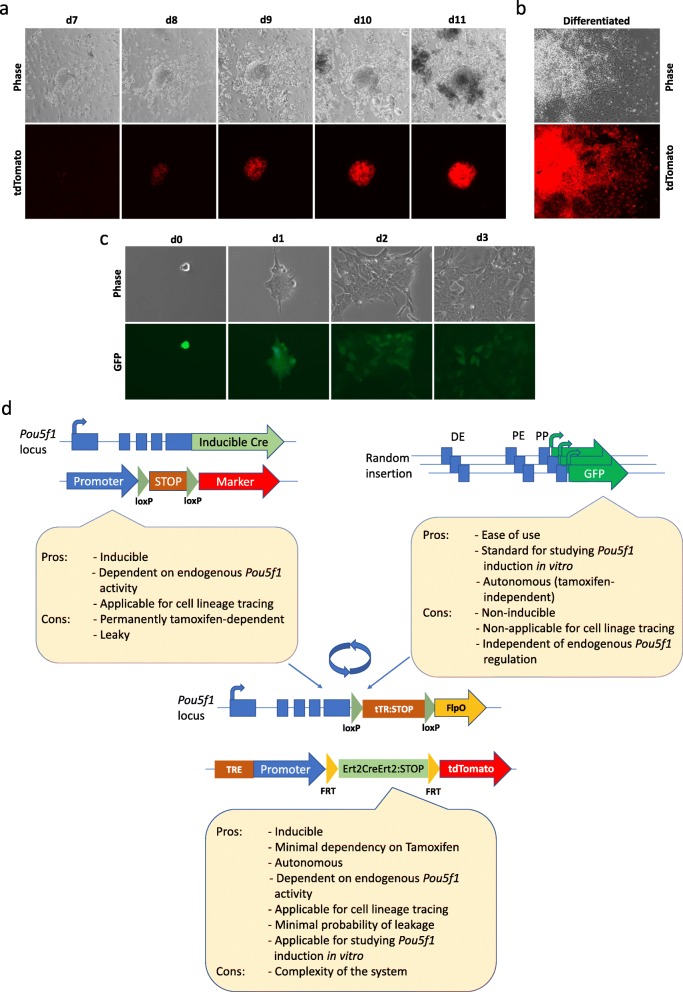
Differentiation and reprogramming of ESCs harboring the developed *Oct4* tracing system in comparison with OG2 reporter system. **a** Onset of the tdTomato expression on day 6 due course of iPSC colony formation following Dox/4-OHT treatment and infection of the O4S ESC-derived neural stem cells (NSCs) with the OKSM lentivirus. **b** Retention of tdTomato signal in differentiated cells derived from O4S ESCs. **c** Loss of GFP signal in differentiated cells derived from OG2 ESCs. **d** Schematic comparison of O4S system with two other common types of systems aimed to study *Oct4* activity

Differentiated neural cells derived from O4S ESCs were sensitized by adding 4-OHT and Dox for 2 days and proceeded to reprogramming with the use of lentivirally delivered OKSM (Oct4, Klf4, Sox2, and cMyc) factor cocktail. According to the obtained results, the expression of tdTomato in O4S cells was detectable as early as day 6–7 following the infection with the OKSM (Fig. 3a). This result is consistent with the previous data which showed activation of Oct4 expression on day 5–6 of reprogramming [36].

O4S ESC colonies were next picked, expanded and differentiated by a withdrawal of LIF. In parallel, we differentiated ESCs derived from transgenic mice OG2 harboring an *Oct4-GFP* transgene [37]. Fully in agreement with the design of each reporter system, differentiated progeny of O4S ESCs retained high levels of tdTomato (Fig. 3b), whereas differentiated OG2 ESCs showed an extinction of the GFP signal (Fig. 3c).

## Discussion

In this study, we have developed and assessed properties of a novel Oct4 tracing system (O4S). A comparison of the O4S system with two related and commonly deployed Oct4 tracing systems is summarized in Fig. 3. Considering the system, that relies on an inducible Cre recombinase knocked into *Oct4* locus (Fig. 3d, left), it can be deployed to trace true *Oct4* gene activity with great degree of confidence [36]. However, main disadvantages of such a system is that (1) it requires continuous presence of tamoxifen to capture the onset of *Oct4* activity, (2) it is prone to leakage, likely due to endogenous estrogens. The latter necessitates additional barriers that can reliably block expression of the recombinase until a desired time point. Another popular *Oct4* tracing system, OG2 [37], is easy to operate and it functions in an autonomous fashion, being independent on continuous presence of tamoxifen (Fig. 3d, right). However, this system also has disadvantages such as, for example, lack of capacity for cell lineage tracing. Major advantage of the developed O4S is that it combines useful features of the two systems and, as a result, it can record even transient *Oct4* gene activation events with no dependency on the continuous presence of sensitizers such as tamoxifen. After sensitization past the epiblast stage O4S system can capture *Oct4* gene activation over virtually unlimited period of mouse ontogeny in a fully autonomous fashion. Lastly, the system should demonstrate a gain of sensitivity, because cells that once set Oct4 expression might keep proliferating, providing thereby signal amplification at the level of tissue and organs (Fig. 3d).

Our recent data has provided genetics-based evidences that the functions of the key pluripotency factor Oct4 go well beyond safeguarding mammalian germline and include control of plasticity of the smooth muscle cells (SMCs) during the development of atherosclerotic lesions [18]. We expect that Oct4 role in the adult mouse, which might involve regulation of cellular plasticity, is not limited to the above case but extends to other cell types, tissues, and organs. The mouse line that is currently generated from the O4S ESCs will be indispensable in our efforts to describe these novel Oct4 features.

## Conclusions

**Fig. 4:**
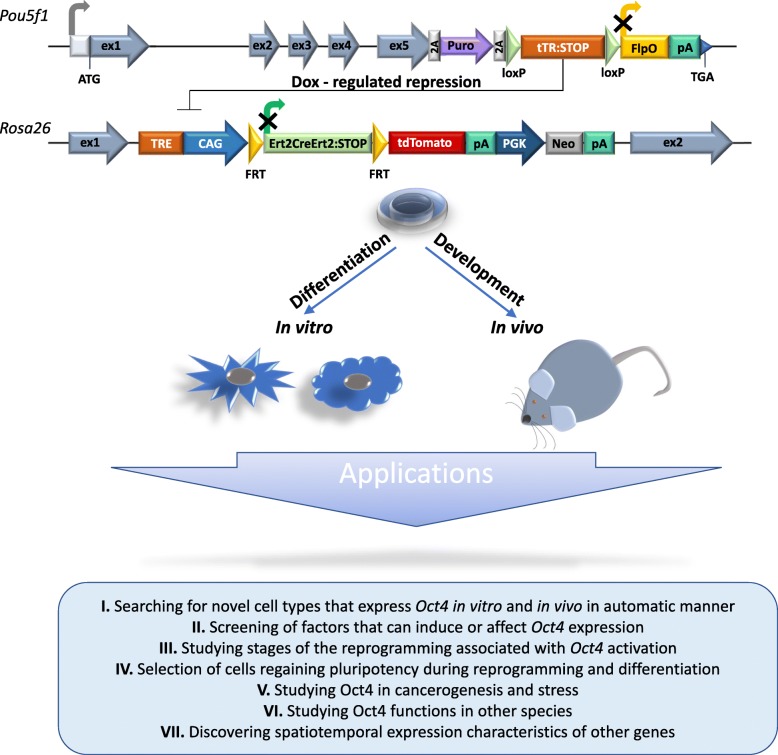
Application of the developed gene tracing approach to monitor the activity of *Oct4* and other genes in cultured cells and during mouse ontogeny with potential biological applications

In our attempt to detect *Oct4* gene expression in vivo and in vitro, we have developed a two-component system that allows tracing the expression of this gene past the epiblast stage of development. Clearly, the developed system can be also applied for different goals, involving examination of *Oct4* expression (Fig. 4). Also, our system can be easily adapted to discovering spatiotemporal expression characteristics of other genes during normal development and/or in response to external stimuli and conditions such as neoplasia, disease onset, stress, etc. Finally, it can be easily adapted to other species as well.

## Supplementary information

**Additional file 1. **OFFtarget analysis of the seven most relevant loci for *Oct4* gRNA (a) and *Rosa26* gRNAs (b).

**Additional file 2.** List of primers used for PCR and sequencing for OFFtarget analysis.

**Additional file 3.** Characterization of differentiated NSC cells and induced pluripotent stem cells. (a) q PCR analysis of the expression of the markers Oct4 and Sox2 in undifferentiated ESCs and differentiated NSC-like cells. Results are expressed as mean +/− SEM (b) Immunocytochemistry against Nestin (green) and Sox2 (red) markers in differentiated NSC. (c) Immunofluorescence staining against Oct4 (Magenta) and Nanog (Green) in the cells with inactive tracing system (left) and cells activating tdTomato expression (right). Note, that cells with bright magenta signals – non-iPSC proliferating cells overexpressing Oct4 during reprogramming procedure.

## Data Availability

All vector constructs can be obtained by contacting the authors.

## Abbreviations

ESC: Embryonic stem cell
iPSC: Induced pluripotent stem cell
4-OHT: 4-hydroxytamoxifen
ERT, Ert2: Estrogen receptor ligand-binding domain
PGC: Primordial germ cell
SMC: Smooth muscle cell
NCS: Neural stem cell
MEF: Mouse embryonic fibroblast
Dox: Doxycycline
tTR: Tetracycline-regulated repressor
TRE: Tetracycline response element
O4S: Oct4 sensor
OKSM: Oct4, Klf4, Sox2, cMyc

## References

[1] Kranz A, Fu J, Duerschke K, Weidlich S, Naumann R, Stewart FA, et al. An improved Flp deleter mouse in C57Bl/6 based on Flpo recombinase. genesis. 2010;48(8):512–20. 10.1002/dvg.20641 20506501

[2] Hsu YC (2015). Theory and practice of lineage tracing. Stem Cells.

[3] Kretzschmar K, Watt FM (2012). Lineage tracing. Cell..

[4] Wang QT, Piotrowska K, Ciemerych MA, Milenkovic L, Scott MP, Davis RW (2004). A genome-wide study of gene activity reveals developmental signaling pathways in the preimplantation mouse embryo. Dev Cell.

[5] Feil R, Wagner J, Metzger D, Chambon P (1997). Regulation of Cre Recombinase activity by mutated estrogen receptor ligand-binding domains. Biochem Biophys Res Commun.

[6] Schöler HR, Balling R, Hatzopoulos AK, Suzuki N, Gruss P (1989). Octamer binding proteins confer transcriptional activity in early mouse embryogenesis. EMBO J.

[7] Schöler HR, Hatzopoulos AK, Balling R, Suzuki N, Gruss P (1989). A family of octamer-specific proteins present during mouse embryogenesis: evidence for germline-specific expression of an Oct factor. EMBO J.

[8] Nichols J, Zevnik B, Anastassiadis K, Niwa H, Klewe-Nebenius D, Chambers I (1998). Formation of pluripotent stem cells in the mammalian embryo depends on the POU transcription factor Oct4. Cell..

[9] Niwa H, Miyazaki J-i, Smith AG. Quantitative expression of Oct-3/4 defines differentiation, dedifferentiation or self-renewal of ES cells. Nature Genetics. 2000;24(4). 10.1038/74199 10742100

[10] Schöler HR, Ruppert S, Suzuki N, Chowdhury K, Gruss P (1990). New type of POU domain in germ line-specific protein Oct-4. Nature..

[11] Radzisheuskaya A, Silva J (2014). Do all roads lead to Oct4? The emerging concepts of induced pluripotency. Trends Cell Biol.

[12] Wu G, Schöler HR (2014). Role of Oct4 in the early embryo development. Cell Regeneration.

[13] Iwafuchi-Doi M, Zaret KS (2014). Pioneer transcription factors in cell reprogramming. Genes Dev.

[14] Takahashi K, Yamanaka S (2006). Induction of pluripotent stem cells from mouse embryonic and adult fibroblast cultures by defined factors. Cell..

[15] Merrell AJ, Stanger BZ (2016). Adult cell plasticity in vivo: de-differentiation and transdifferentiation are back in style. Nat Rev Mol Cell Biol.

[16] Mitchell RR, Szabo E, Benoit YD, Case DT, Mechael R, Alamilla J (2014). Activation of neural cell fate programs toward direct conversion of adult human fibroblasts into tri-potent neural progenitors using OCT-4. Stem Cells Dev.

[17] Szabo E, Rampalli S, Risueño RM, Schnerch A, Mitchell R, Fiebig-Comyn A (2010). Direct conversion of human fibroblasts to multilineage blood progenitors. Nature..

[18] Cherepanova OA, Gomez D, Shankman LS, Swiatlowska P, Williams J, Sarmento OF (2016). Activation of the pluripotency factor OCT4 in smooth muscle cells is atheroprotective. Nat Med.

[19] Lengner CJ, Camargo FD, Hochedlinger K, Welstead GG, Zaidi S, Gokhale S (2007). Oct4 expression is not required for mouse somatic stem cell self-renewal. Cell Stem Cell.

[20] Sommer CA, Stadtfeld M, Murphy GJ, Hochedlinger K, Kotton DN, Mostoslavsky G (2009). Induced pluripotent stem cell generation using a single lentiviral stem cell cassette. Stem Cells.

[21] Hong YJ, Kim JS, Choi HW, Song H, Park C, Do JT (2016). In vivo generation of neural stem cells through teratoma formation. Stem Cells Dev.

[22] Conti L, Pollard SM, Gorba T, Reitano E, Toselli M, Biella G (2005). Niche-independent symmetrical self-renewal of a mammalian tissue stem cell. PLoS Biol.

[23] Wiznerowicz M, of virology T-D. Conditional suppression of cellular genes: lentivirus vector-mediated drug-inducible RNA interference. Journal of virology. 2003. 10.1128/JVI.77.16.8957-8961.2003 PMC167245 12885912

[24] Liskovykh M, Chuykin I, Ranjan A, Safina D, Popova E, Tolkunova E, et al. Derivation, characterization, and stable transfection of induced pluripotent stem cells from Fischer344 rats. PLOS one. 2011;6(11). 10.1371/journal.pone.0027345 PMC3208629 22076153

[25] Malashicheva A, Kanzler B, Tolkunova E, Trono D (2007). Tomilin A. Lentivirus as a tool for lineage-specific gene manipulations genesis.

[26] Sinenko SA, Skvortsova EV, Liskovykh MA, Ponomartsev SV, Kuzmin AA, Khudiakov AA (2018). Transfer of synthetic human chromosome into human induced pluripotent stem cells for biomedical applications. Cells..

[27] Skvortsova EV, Sinenko SA, Tomilin AN (2018). Immortalized murine fibroblast cell lines are refractory to reprogramming to pluripotent state. Oncotarget..

[28] Raymond CS, Soriano P. High-efficiency FLP and C31 site-specific recombination in mammalian cells. PloS one. 2007;2(1). 10.1371/journal.pone.0000162 PMC1764711 17225864

[29] Chen C-mM, Krohn J, Bhattacharya S, Davies B. A comparison of exogenous promoter activity at the ROSA26 locus using a ΦiC31 integrase mediated cassette exchange approach in mouse ES cells. PloS one. 2011;6(8). 10.1371/journal.pone.0023376 PMC3154917 21853122

[30] Tchorz JS, Suply T, Ksiazek I, Giachino C, Cloëtta D, Danzer C-PP, et al. A modified RMCE-compatible Rosa26 locus for the expression of transgenes from exogenous promoters. PloS one. 2012;7(1). 10.1371/journal.pone.0030011 PMC3258265 22253858

[31] Matsuda T, Cepko CL (2007). Controlled expression of transgenes introduced by in vivo electroporation. Proc Natl Acad Sci U S A.

[32] Szulc J, Wiznerowicz M, Sauvain M-O, Trono D, Aebischer P. A versatile tool for conditional gene expression and knockdown. Nature Methods. 2006;3(2). 10.1038/nmeth846 16432520

[33] Hsu PD, Lander ES, Zhang F (2014). Development and applications of CRISPR-Cas9 for genome engineering. Cell..

[34] Szymczak AL, Workman CJ, Wang Y, Vignali KM, Dilioglou S, Vanin EF (2004). Correction of multi-gene deficiency in vivo using a single ‘self-cleaving’ 2A peptide-based retroviral vector. Nat Biotechnol.

[35] Ellis P, Fagan BM, Magness ST, Hutton S, Taranova O, Hayashi S (2004). SOX2, a persistent marker for multipotential neural stem cells derived from embryonic stem cells, the embryo or the adult. Dev Neurosci.

[36] Greder LV, Gupta S, Li S, Abedin MJ, Sajini A, Segal Y, et al. Analysis of endogenous Oct4 activation during induced pluripotent stem cell reprogramming using an inducible Oct4 lineage label. Stem cells (Dayton, Ohio). 2012;30(11):2596–601. 10.1002/stem.1216 PMC3626284 22948941

[37] Szabó PE, Hübner K, Schöler H, Mann JR (2002). Allele-specific expression of imprinted genes in mouse migratory primordial germ cells. Mech Dev.

