# Supplementary material for: Genetic tool for fate mapping of Oct4 (Pou5f1)-expressing cells and their progeny past the pluripotency stage
Source: Stem Cell Res Ther. 2019 Dec 16;10:391. doi: 10.1186/s13287-019-1520-6 (PMC6916430; doi:10.1186/s13287-019-1520-6)

a

## Oct4 OFFtarget analysis

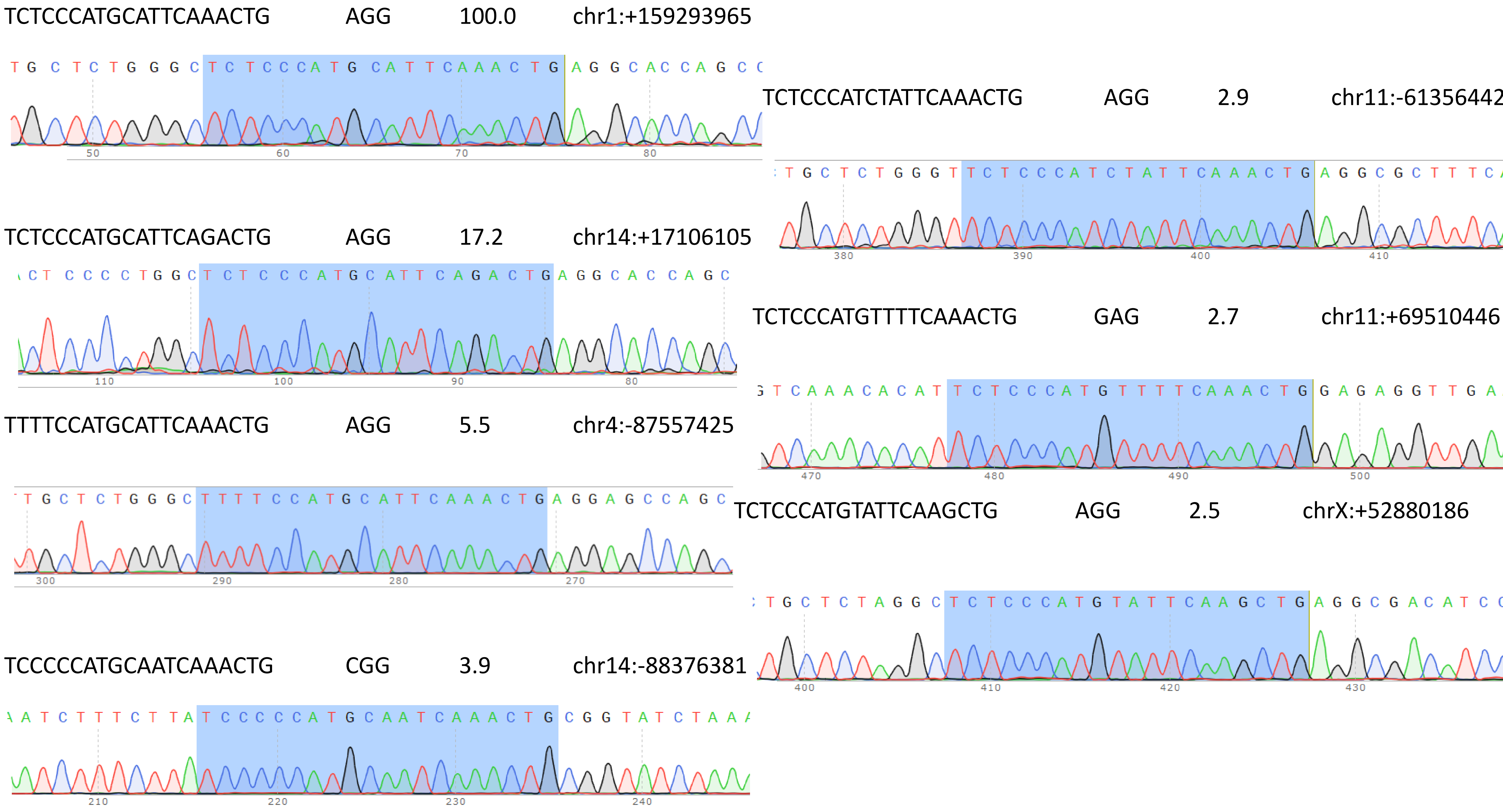


b

## Rosa26 OFFtarget analysis

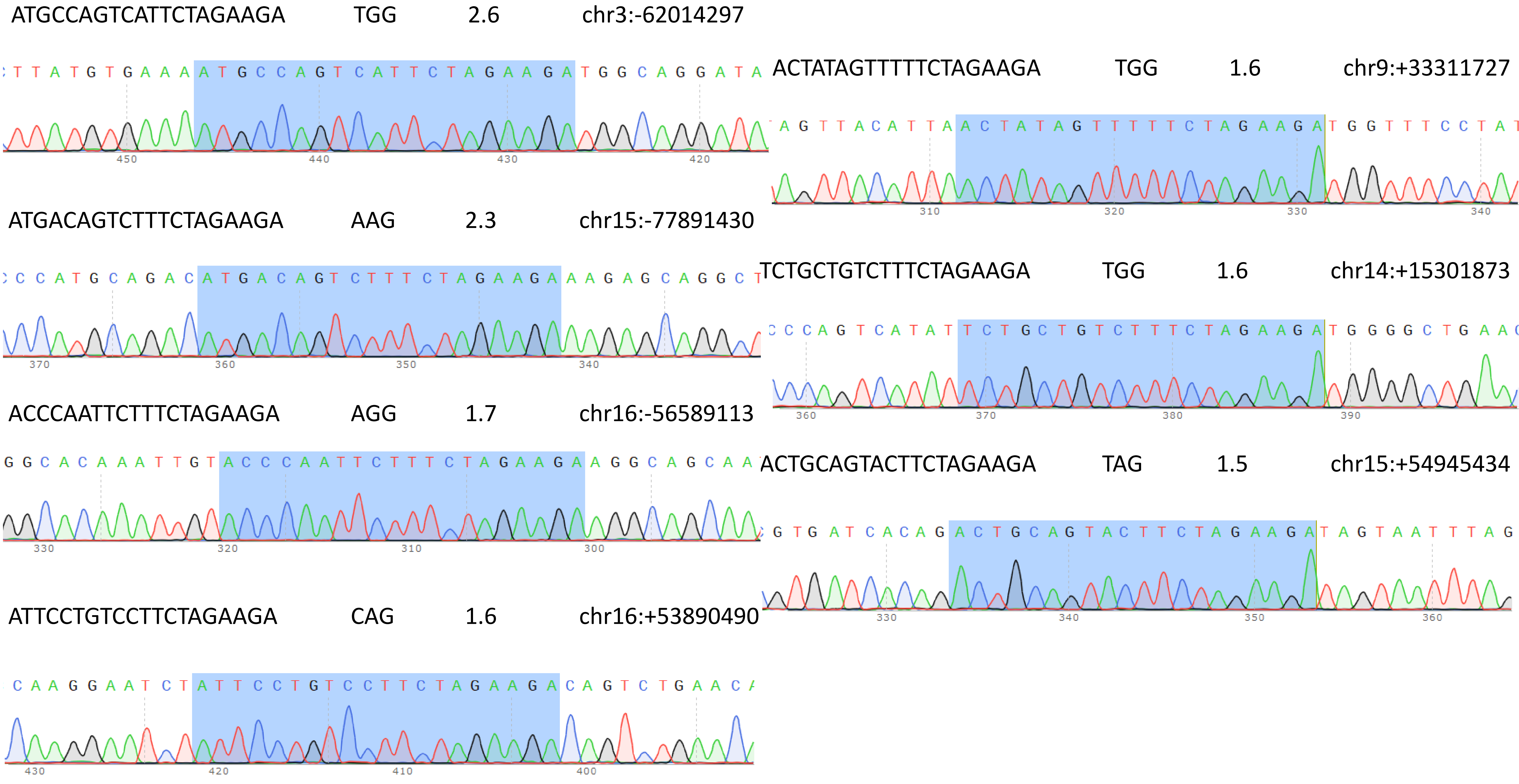

Supplement: Supplementary file 1 — Additional file 1. OFFtarget analysis of the seven most relevant loci for Oct4 gRNA (a) and Rosa26 gRNAs (b). [file 13287_2019_1520_MOESM1_ESM.pdf]
