# Supplementary figures and images for: Genetic tool for fate mapping of Oct4 (Pou5f1)-expressing cells and their progeny past the pluripotency stage
Source: Stem Cell Res Ther. 2019 Dec 16;10:391. doi: 10.1186/s13287-019-1520-6 (PMC6916430; doi:10.1186/s13287-019-1520-6)

a

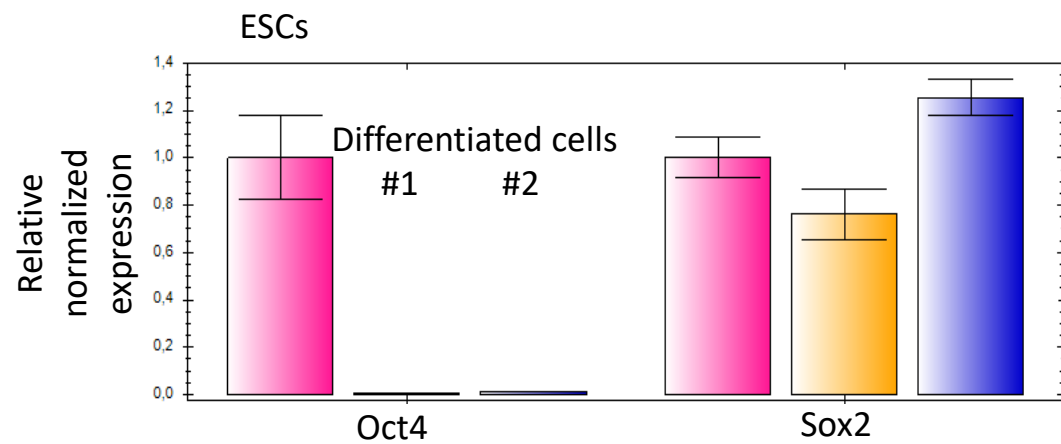

b

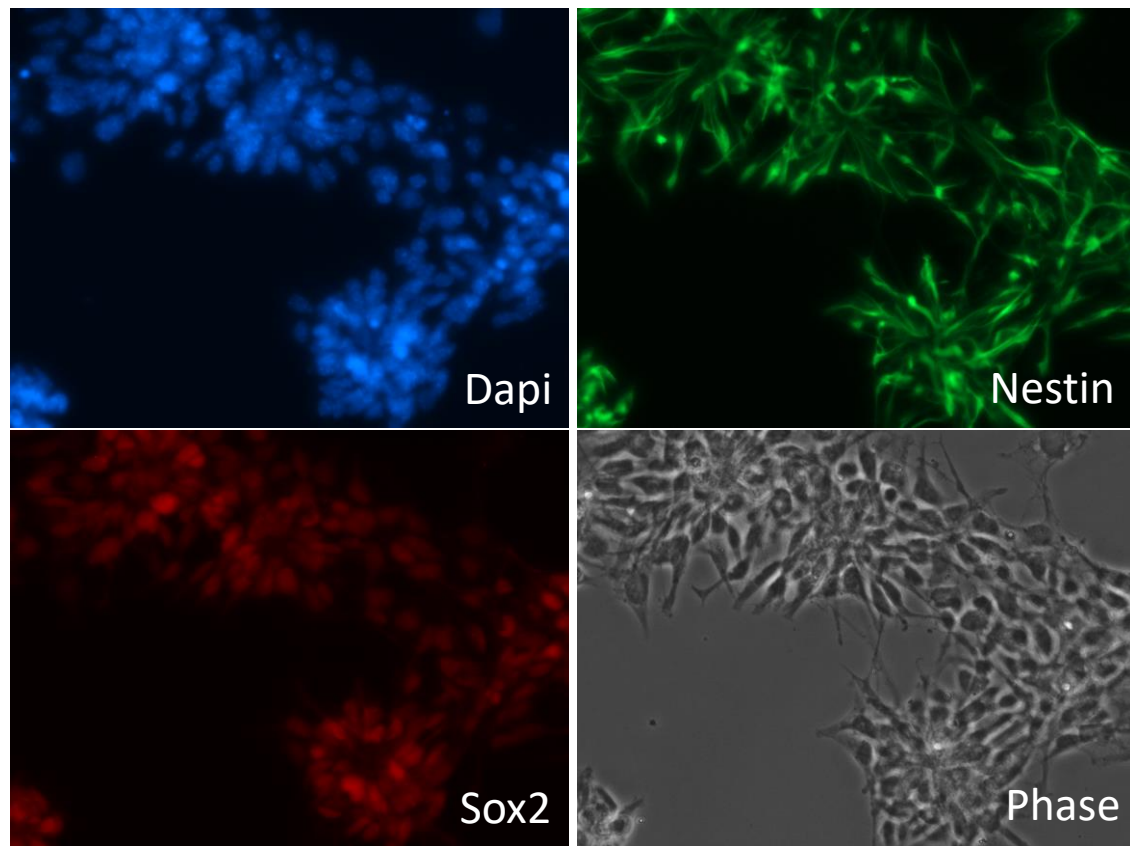

c

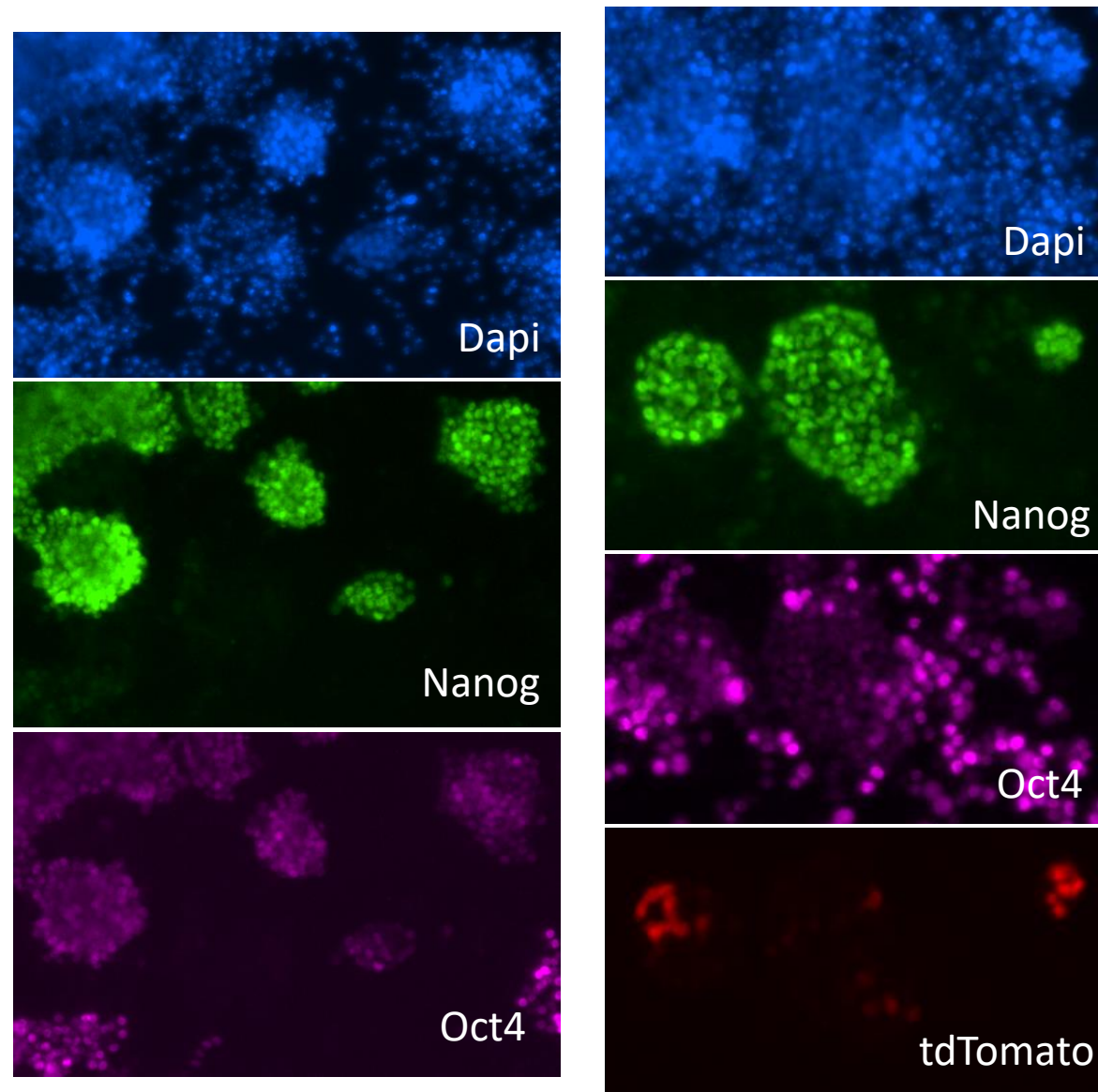

Supplement: Supplementary file 2 — Additional file 2. List of primers used for PCR and sequencing for OFFtarget analysis. [file 13287_2019_1520_MOESM2_ESM.pdf]
